# The role of inflammatory processes and zinc levels in prostatic enlargement among Iraqi samples

**DOI:** 10.25122/jml-2023-0224

**Published:** 2023-09

**Authors:** Ali Mohammed Sameen, Ibraheem Abdulnabi Shabeeb, Rashied Mohammed Rashied

**Affiliations:** 1 Department of Biology, College of Science, University of Anbar, Ramadi, Iraq

**Keywords:** PSA, IL6, IL8, Zinc, CRP, WBC count, AUR: Acute Urinary Retention, BPH: Benign Prostatic Hyperplasia, CRP: C-Reactive Protein, ESR: Erythrocyte Sedimentation Rate, IL-6: Interleukin-6, IL-8: Interleukin-8, MCP-1: Monocyte Chemoattractant Protein-1, NLR: Neutrophil-Lymphocyte Ratio, PC: Prostate Cancer, PSA: Prostate-Specific Antigen, PLR: Platelet-Lymphocyte Ratio, SIL-8: Serum Interleukin-8, WBC: White Blood Cell Count

## Abstract

This study aimed to investigate the role of inflammatory processes in benign prostatic enlargement among men with elevated prostate-specific antigen (PSA) levels without a history of prostatic disease. Additionally, we aimed to examine the influence of serum zinc levels on prostate volume. We investigated the associations between systemic inflammatory markers, serum PSA, and serum zinc levels in 48 men without a history of prostatic disease, aged between 60-72 years, and 30 healthy men in the same age range. Data collection occurred between 1/2/2022 to 1/10/2022. The results are presented as mean values ± standard error (SE), and statistical significance was determined at p≤0.05. The levels of sIL-8 (P: 44.295±1.002, C: 1.404±0.2562), IL-6 (P: 7.406±0.5632, C: 4.468±0.830), CRP (P: 14.765±0.565, C: 6.267±0.538), increased significantly in patients with high PSA, while zinc levels (P: 92.305±2.8235, C: 114.565±8.861) decreased in the patient group. Regarding white blood cell (WBC) parameters, patients exhibited a significant increase in WBC total count (P: 12995.00±488.47, C: 7713.333±777.778), neutrophil % (P: 69.450±1.619, C: 51.200±1.826), lymphocyte % (P: 39.50±2.024, C: 30.867±1.268), and NLR (2.013±0.105). Conversely, there were no significant differences in eosinophil % (P: 3.450±0.4558, C: 3.267±0.5297), basophil % (P: 0.300±0.105, C: 0.267±1182), or monocyte % (P: 3.450±0.4558, C: 3.267±0.5297) between the two groups. In men without known prostatic illness, increased PSA was linked to markers of systemic inflammation. The results indicate the role of inflammatory processes in increasing the size of the prostate gland, as evidenced by the increased levels of immune markers like white blood cells and interleukins, along with the influence of zinc. Future research is required to determine how these markers relate to the development and incidence of prostate cancer.

## INTRODUCTION

The serum prostate-specific antigen (PSA) test remains the most widely utilized screening tool for prostate cancer (PC), although its value is still debatable. While PSA has strong sensitivity, it has low specificity since it is challenging to distinguish between patients with PC and those with benign prostatic illnesses [1]. High PSA levels have been associated with urinary tract infections, acute or chronic prostatitis, or benign prostatic hyperplasia (BPH) [2]. However, various conditions connected to the prostate, including prostatitis, BPH, and other treatments, can increase serum PSA levels. It is important to note that prior studies [2] have mostly focused on PSA concentrations of 4.0 ng/mL or higher. Nowadays, it is thought that inflammation contributes to the progression and development of prostate issues [3-7] and that chronic inflammation is present in more than 75% of BPH surgical specimens [8].

Research has examined the relationship between systemic inflammation and PSA characteristics. Several disorders linked to increased PSA levels are related to systemic inflammation [9]. Nevertheless, the relationship between systemic inflammation and PSA levels in asymptomatic males is unknown. This is significant because identifying indicators corresponding to PSA levels and tracking PC risk could become a valuable additional tool to evaluate PC risk in men with elevated PSA [10]. A higher risk of developing colorectal cancer is linked to high levels of circulating C-reactive protein (CRP) and erythrocyte sedimentation rate (ESR) [11]. Lung cancer risk is inversely correlated with CRP and ESR levels [12]. The development of PC is linked to CRP, neutrophil count, platelet-lymphocyte ratio (PLR), and neutrophil-lymphocyte ratio (NLR) [13]. Low-grade systemic inflammation is correlated with high white blood cell count (WBC) and CRP readings. Moreover, carotid atherosclerosis is linked to higher WBC counts and CRP levels [14]. Mild systemic inflammation, characterized by elevated WBC and CRP levels, has been associated with obesity and diabetes [15].

Lately, some articles have connected inflammation and subclinical PSA levels. In order to best assist asymptomatic patients with increased PSA levels, systemic inflammation should be examined. Markers of systemic inflammation have been the subject of several further research. However, most of those articles have concentrated on PSA levels of 4.0 ng/mL or higher [16].

Inflammation, both locally and systemically, has been researched to comprehend the biological significance of high PSA values. A powerful biomarker for diagnosing prostate inflammatory diseases, such as BPH and chronic pelvic pain syndrome/chronic prostatitis, has been identified as interleukin 8 (IL-8) in seminal plasma [17]. However, there is little evidence that serum IL-8 (sIL-8) can predict the risk of patients with high PSA levels [18]. Numerous healthy cells produce the multipurpose cytokine interleukin-6 (IL-6), such as monocytes, fibroblasts, and T lymphocytes. Moreover, IL-6 promotes the growth of numerous malignancies, such as prostate cancer, melanoma, renal cell carcinoma, ovarian carcinoma, and lymphoma [19, 20]. Pro-inflammatory cytokines were also linked to PSA generation and prostate cancer development [21]. Zinc is necessary for cell development and reproduction and constituent of many metalloenzymes. The prostate has ten times more total zinc than other soft tissues do. Prostate epithelial cells have a quick uptake of zinc under physiological conditions of testosterone and prolactin levels, and the transportation process is facilitated by a cell membrane transporter. On the other hand, the ability of prostate tumor adenocarcinoma cells to accumulate zinc is lost [22]. The purpose of this study was to investigate the impact of inflammatory processes, as indicated by specific immunological markers, on prostate enlargement. Additionally, the study aimed to explore the role of zinc in this context.

## MATERIAL AND METHODS

We enrolled men aged 60 to 72 recently diagnosed with prostate enlargement and had not undergone previous PSA testing. Recruitment occurred from January 2, 2022, to January 10, 2022, resulting in a cohort of 48 individuals with PSA levels exceeding 4 ng/ml. In addition, we collected blood samples from 30 healthy men within the same age range, and patients who had a differential white blood cell count test were compared with them. The prostate antigen test was carried out after the sample was centrifuged to extract the serum. The exclusion criteria included chronic illnesses and medication use.

Automated analyzers were used to evaluate laboratory tests that included neutrophil-lymphocyte ratio (NLR), differential white cell count (neutrophils, lymphocytes, basophils, eosinophils, monocytes), and white blood cell count (WBC). C-reactive protein was measured using particle-enhanced immunoturbidimetric analysis (Roche C-Reactive Protein Latex, COBAS) following standardized protocols. Serum total PSA, interleukin-6 (IL-6), and interleukin-8 (IL-8) assays were performed using the Fine test (Wuhan Fine Biotech). Serum trace element zinc levels were measured using the Genotek fully automated Zinc Giesse Kit (Giesse diagnostic - Italy), which involved a deproteinization process.

### Statistical analysis

Statistical analyses were conducted using Microsoft Excel 2016, Statistical Package for the Social Sciences 19.0 (SPSS Inc., Chicago, IL, USA), and GraphPad Inc. (San Diego, CA, USA). Continuous variables were presented as the mean and standard error (SE) [23, 24].

## RESULTS

**Figure 1:**
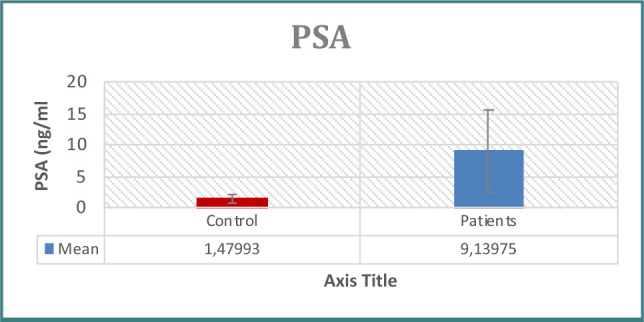
PSA levels across groups

**Figure 2:**
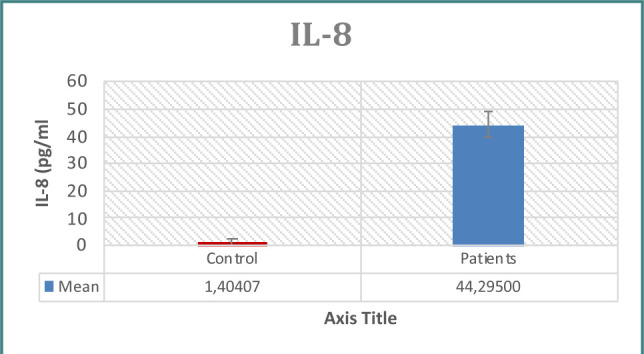
IL-8 levels in study groups

**Figure 3:**
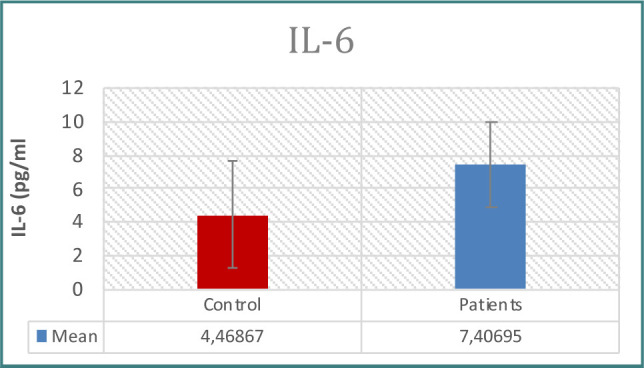
IL-6 levels in study groups

**Figure 4:**
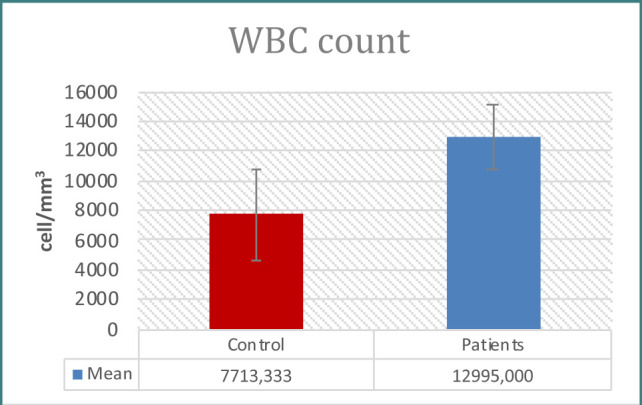
WBC count in study groups

**Figure 5:**
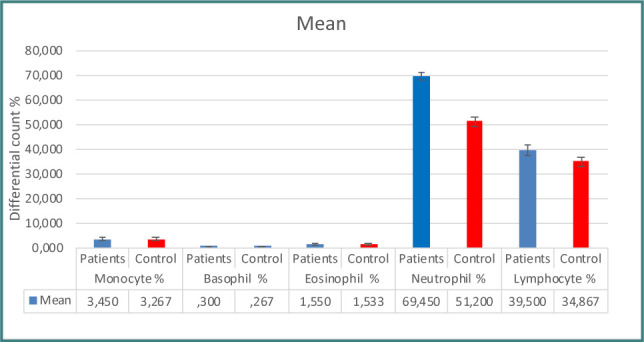
Differential WBC count in study groups

**Figure 6:**
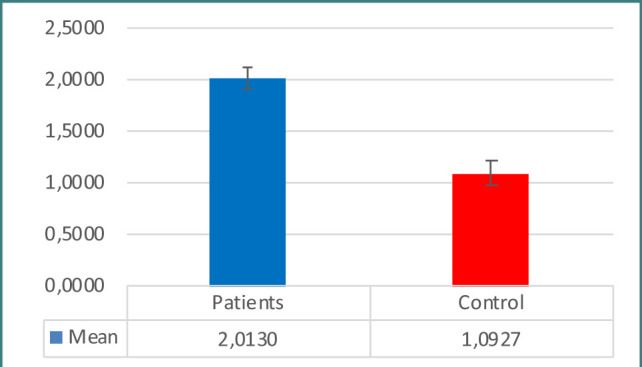
NLR ratio in study groups

**Figure 7:**
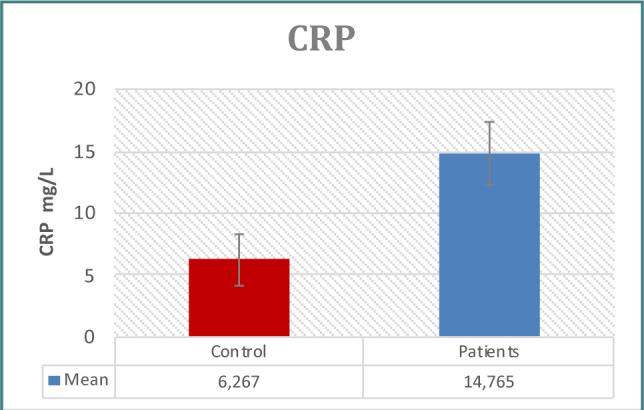
CRP levels in study groups

Patients with prostate issues had significantly higher PSA concentrations than controls, as seen in Table 1 and Figure 1. When examining immunological indicators, the results for IL-6, IL-8, WBC count, WBC differential count (neutrophils, lymphocytes), CRP, and NLR revealed higher levels in the patient group, demonstrating statistically significant differences between the two groups (Tables 1-2 and Figures 2-7). However, the variations in monocyte, eosinophil, and basophil levels between the groups were not statistically significant (p<0.05).

**Table 1:** Comparative analysis of PSA, IL-8, IL-6, CRP, and zinc levels between patients and control group

Parameters	Patients	Control
PSA IL-8 IL-6 CRP Zinc	9.1397±1.474 44.295±1.002 7.406±0.5632 14.765±0.565 92.305±2.8235	1.4799±0.19995 1.404±0.2562 4.468±0.830 6.267±0.538 114.565±8.861

* p≤0.05

**Table 2:** Comparison of white blood cell (WBC) parameters between patients and control group

WBC Parameters	Patients	Control
WBC count Neutrophil % Eosinophil % Basophil % Monocyte % Lymphocyte % NLR	12995.00±488.47 69.450±1.619 1.550±0.2562 0.300±0.105 3.450±0.4558 39.50±2.024 2.013±0.105	7713.333±777.778 51.200±1.826 1.533±0.3362 0.267±1182 3.267±0.5297 30.867±1.268 1.092±0.128

* p≤0.05

**Figure 8:**
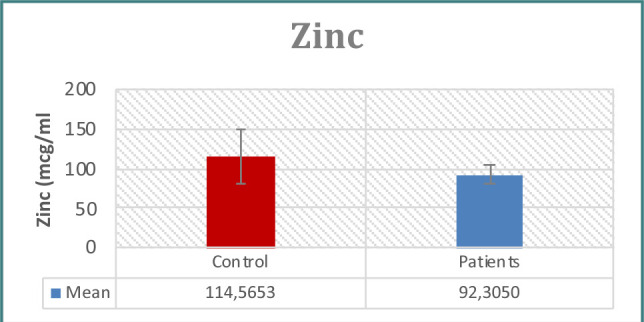
Zinc levels in study groups

Regarding zinc levels, patients had a decrease compared to the control group (p<0.05) (Figure 8).

## DISCUSSION

The current study is, to our knowledge, the first to date to investigate the association between blood PSA levels in males without prior systemic inflammation and a number of inflammatory indicators. The study obtained PSA data from a clinical laboratory, including 48 men with PSA screenings. Consequently, some might have had PC, PBH, prostatitis, or acute urine retention (AUR). PSA is still one of the most effective markers for early prostate diagnosis issues [25]. Using a PSA cut-off value of 4 ng/mL, this study looked into the relationship between serum PSA levels and inflammatory indicators in the body. In our research, we discovered a link between prostatic inflammation and peripheral WBC count. This discovery supports the results of earlier research by Dr. Kazutoshi *et al*. [26], which showed a correlation between peripheral WBC count and the severity of BPH. Hence, it may be inferred that a higher peripheral WBC count indicates more systemic inflammation, which exacerbates prostatic inflammation and worsens BPH.

Moreover, we discovered a favorable correlation between the peripheral neutrophil and lymphocyte count and prostatic inflammation, demonstrating the inherent relationship between systemic and prostatic inflammation [27]. While the mechanisms driving prostate development in individuals with elevated WBC counts are not fully understood, hypotheses suggest that neutrophils may recruit monocytes by interacting with endothelial cells, leading to the release of Monocyte Chemoattractant Protein-1 (MCP-1) [28]. In the general population, an increased serum MCP-1 level is linked to a high WBC count [29]. In vitro, MCP-1 attracts monocytes and stimulates prostate epithelial cells, and it is secreted by prostate stromal cells in response to inflammatory cytokines [30].

Another possibility is that active neutrophils damage endothelial cells through increased release of the enzyme elastase. Hypoxia-stimulated prostate development could result from the aberrant blood flow that develops around the damaged vasculatures in the prostate [31]. Prior studies showed that high serum PSA and high NLR are related [32]. Lymphocytes are involved in adaptive immunity, while neutrophils are involved in innate immunity. NLR is an indicator of the balance between neutrophils and lymphocytes [33]. In this study, NLRs were higher among men with elevated serum PSA levels compared to those with normal PSA levels. The relationship between NLR and elevated blood PSA levels may indicate a problem with the adaptive ability of the host to control inflammation, as NLR reflects the equilibrium between adaptive (lymphocytes) immune responses and innate (neutrophils) responses [32-35].

In the current study, individuals with elevated blood PSA levels had higher serum CRP levels than men with normal serum PSA. This result is in line with a study that discovered a positive correlation between serum CRP and PSA in males (n=302) who were over 35 and sent for PC screening, as well as in men with PSA levels greater than 2.5 ng/ml [36]. Two investigations found a link between PC and serum CRP, one in the Doctors' Health Study and the other among a small sample of 156 French men [37]. Further research is needed to determine whether males with elevated PSA and CRP have a higher chance of developing PC [38].

Interleukin 8 is a pro-inflammatory Cysteine-X-Cysteine (CXC) chemokine that contributes to the inflammation connected with BPH and other inflammatory diseases [39, 40]. Cross-sectional studies of sIL-6 and sIL-8 in 256 and 245 BPH patients were conducted using univariate and multivariate logistic regression analyses. The results revealed a positive correlation between sIL-6 and sIL-8 levels in the AUR group and AUR [41, 42].

Another study evaluated the expressions of IL-8 in patients with BPH and those with BPH accompanied by prostatic inflammation and revealed a significantly higher occurrence of urine retention among those whose condition was aggravated by inflammation compared to those with BPH alone. Wu *et al*. confirmed the high level of interleukin in the serum of patients with benign prostatic hyperplasia who suffer from acute urinary retention, which can be used as an early predictor of acute urinary retention. Significant cytokines that play an important role in the immunological inflammation of prostate hyperplasia include IL-6 and IL-8, found in prostatic fluid. Numerous research have examined the connection between the prevalence of acute urine retention (AUR) in PBH patients and plasma levels of serum IL-6 and IL-8 [43-45].

Numerous laboratory investigations have consistently highlighted the significance of impaired zinc retention as a critical factor in the proliferation and progression of malignant prostate cells. Prostate epithelial cells contain extremely high zinc concentrations in their mitochondria, where zinc suppresses citrate oxidation and inhibits mitochondrial aconites. Malignant prostate cells, it turns out, have a higher rate of citrate oxidation than normal prostate cells, although increased citrate oxidation is not currently believed to cause prostate carcinogenesis [18].

Incubating prostate cancer cell lines with physiological levels of zinc led to a noticeable suppression of cell development, according to Liang's experiments [46]. Our investigation supported the findings of Li *et al*., which established that men with high PSA values have lower blood levels of zinc than men with normal PSA values and that this difference serves as a prostate cancer risk factor [47].

## CONCLUSION

In the context of prostate cancer management, continuous patient monitoring and the identification of associated risk factors hold significant importance. Our study showed an increase in various indicators associated with inflammatory pathways in individuals diagnosed with benign prostatic hyperplasia compared to healthy individuals in Iraq. There were elevated levels of interleukin IL-8 and IL-6, as well as an increase in prostate-specific antigen among individuals affected by prostatic hyperplasia. Additionally, there was an increase in circulating C-reactive protein (CRP) levels and total leukocyte count. Zinc levels were lower in affected individuals compared to healthy people.
